# Bathing during the Menstrual Period

**Published:** 1905-04

**Authors:** 


### Abstracts and Selections.

### Bathing During the Menstrual Period.

Edgar concludes in the *American Journal of Obstetrics* that all forms of bathing during the menstrual period are largely a matter of habit, and usually can be acquired by cautious and gentle progression, but not for every woman does this hold good, and surf bathing, where the body surface remains chilled for some time, should always be excepted.

A daily tepid sponge bath (85° to 92° F.) during the menstrual period is not only a harmless proceeding, but is demanded by the rules of hygiene.

In the majority, if not all women, tepid (85° to 92° F.) sponge bathing after the establishment of the menstrual flow, namely, second or third day, is a perfectly safe practice.

Further, in most women the habit of using the tepid shower or tub bath after the first day or two of the flow can with safety be acquired.

